# Crowd-sourced investigation of a potential relationship between *Bartonella*-associated cutaneous lesions and neuropsychiatric symptoms

**DOI:** 10.3389/fpsyt.2023.1244121

**Published:** 2023-10-24

**Authors:** Zachary Stewart, Sanvi Korsapathy, Flavio Frohlich

**Affiliations:** ^1^Department of Psychiatry, University of North Carolina at Chapel Hill, Chapel Hill, NC, United States; ^2^Carolina Center for Neurostimulation, University of North Carolina at Chapel Hill, Chapel Hill, NC, United States; ^3^Neuroscience Center, University of North Carolina at Chapel Hill, Chapel Hill, NC, United States; ^4^Department of Cell Biology and Physiology, University of North Carolina at Chapel Hill, Chapel Hill, NC, United States; ^5^Department of Biomedical Engineering, University of North Carolina at Chapel Hill, Chapel Hill, NC, United States; ^6^Department of Neurology, University of North Carolina at Chapel Hill, Chapel Hill, NC, United States

**Keywords:** *Bartonella*, depression, anxiety, schizotypy, MTurk

## Abstract

**Introduction:**

Preliminary studies suggest that infection with Bartonella bacteria can not only cause a characteristic rash, headache, fever, and fatigue but also neuropsychiatric symptoms. To date, this association has only been reported in case studies, and it remains unclear if this association generalizes to larger samples.

**Methods:**

We used Amazon's Mechanical Turk (MTurk) to crowdsource a large sample (N = 996) of individuals to ascertain the extent to which the presence of participant-identified Bartonella-associated cutaneous lesions (BACL) was associated with self-reported measures of anxiety, depression, and schizotypy. Participants were asked to select images of cutaneous lesions they had seen on their own bodies and complete a battery of self-report questionnaires to assess psychiatric symptoms. Participants were not informed that the focus of the study was on potential dermatological lesions associated with Bartonella. Point-biserial correlations were used to determine the potential relationship between selecting a BACL image and the severity of self-reported psychiatric symptoms.

**Results:**

Scores of anxiety, depression, and schizotypy were positively and significantly correlated with selecting a BACL image. Furthermore, self-report scores of 10 or higher on the GAD-7 and PHQ-9, which represent the suggested clinical cutoffs for meeting criteria for a depressive or anxiety-related disorder, were also significantly associated with selecting a BACL image. Non-Bartonella-associated cutaneous legions were also significantly associated with self-reported measures of psychiatric symptoms.

**Discussion:**

The current study broadens the link between the presence of BACL and the presence of psychiatric symptoms of anxiety, depression, and schizotypy and extends a potential relationship beyond the small sample sizes of previous case studies and case series. Further investigation is recommended to address limitations and expand on these findings.

## Introduction

*Bartonella* is a genus of gram-negative bacteria that can cause a range of diseases in humans and animals (1). Although *Bartonella* infections are more frequently found in animals, they can be transmitted to humans through fleas or cats (2). Several different strains of *Bartonella* bacteria have been linked to human disease, which is often characterized by headaches, fever, and fatigue, and cutaneous lesions such as rashes (1, 2).

Several case studies reported the onset of psychiatric symptoms that coincided with Bartonellosis and associated rashes (3, 4). One such case series, documenting almost 30 individuals with self-reported psychiatric symptoms, had 83% of the sample report cutaneous lesions that appeared with psychiatric symptom onset (5). In another study, symptoms of major depressive disorder, panic attacks, generalized anxiety disorder, and social anxiety, which were unresponsive to standard courses of psychiatric pharmacological treatment, abated with the administration of antibiotics to treat suspected *Bartonella* infections (6). A case-control study found that individuals with schizophrenia/schizoaffective disorder were more likely to test positive on a sensitive PCR assay compared to control participants (7). Overall, this previous research investigating a link between *Bartonella* infection and psychiatric symptoms is limited to case studies or case series with small samples. The degree of available evidence is thus low in quantity and quality, as there are no large studies that are adequately powered and designed to demonstrate a causal link between infection with *Bartonella* and the emergence of psychiatric symptoms. To start to address this gap, we used a crowd-sourced platform to examine the relative prevalence and association of self-reported psychiatric symptoms and the identification of past or current cutaneous lesions associated with *Bartonella* in a large, easy-to-access participant sample.

Amazon's Mechanical Turk (MTurk) has been widely used in research, with investigators seeking to utilize the ability to crowdsource large samples quickly. Researchers submit a Human Intelligence Task (HIT) to the platform, which can be completed by MTurk workers (Turkers). Tasks can be designed within the MTurk space, or templates found on MTurk can be used to link to outside survey platforms such as Qualtrics. The use of MTurk in social science and medical research has been the topic of some controversy over the years, but overall, it is an acceptable and effective method of data collection when used ethically and with outlined best practices (8, 9). Best practices for the use of MTurk have been the recent focus of research to leverage MTurk's ability to rapidly recruit large, diverse samples. Emerging guidelines include procedures for the use of attention checks, screening for bots, ensuring location accuracy, preventing fraudulent responses, and ensuring task completion through methods such as screenshots or completion codes (8–10). To improve data quality, built-in MTurk functionality can be used to only allow certain Turkers to complete a given HIT, using qualifications such as the number of HITs completed, location, and HIT approval rating (10).

The present study aimed to (1) examine the relationship between the presence of BACL and psychiatric symptoms within an MTurk sample and (2) explore the relationship between the presence of other skin manifestations and psychiatric symptoms. Our hypothesis for aim 1 was that there would be a significant relationship between the presence of BACL and psychiatric symptoms of anxiety, depression, and schizotypy. We had no prior hypothesis for aim 2 but rather intended to use images of other skin manifestations as a type of pseudo-control and to protect respondents from determining the study's purpose.

## Methods

### Study design

This is an observational study that was conducted remotely by researchers at the University of North Carolina at Chapel Hill. It was approved by the non-biomedical UNC-Chapel Hill Institutional Review Board (IRB#21-2863). We also conceptualized *a priori, post-hoc* pseudo case-control comparisons. For the purpose of this study, a case was defined as an individual who identified an image of a BACL as something they had previously seen on their own body. The control group was defined as individuals who did not select *Bartonella*-associated images. To control for the potential effect of group size differences, random sampling was used to pull an equal number of data points from each group for statistical comparison. Original analyses using different group sizes were ultimately used as they produced similar results.

### Participants

A total of 996 participants were recruited using a crowd-sourced convenience sampling method via MTurk. A Human Intelligence Task (HIT) was posted on MTurk with a brief description of the study and a link to our study questionnaire on Qualtrics. The first item in the battery of surveys included an information sheet that asked participants to consent to enrollment in the study. Embedded within the demographics section of the study questionnaire were data fidelity checks designed to eliminate responses by BOTs, individuals outside the United States accessing the HIT via a Virtual Private Network (VPN), and those not paying close attention to their responses. Quality control checks proposed by Agley et al. in previous research were used to ensure data quality (10). Participants who did not consent to participation or who did not correctly respond to any of the quality control questions had their survey immediately terminated and were instructed to return the HIT to avoid it being rejected.

### Measures and outcomes

Participants were redirected from the MTurk HIT to our study questionnaire hosted on Qualtrics. The study questionnaire consisted of a Demographics section, in which the data fidelity questions were embedded to ensure the quality of the data. There were four data fidelity questions, each designed to address the data quality concerns outlined in previous research (10). Data fidelity questions focused on attentiveness, and BOT and VPN checks. If a respondent incorrectly answered a data fidelity question, they were automatically skipped to the end of the questionnaire, directed to return the HIT to avoid their work being rejected, and were not compensated for their response. Data fidelity questions were concentrated toward the beginning of the survey to avoid collecting primary outcome data from respondents that would ultimately be excluded.

To assess the presence of BACL, the investigators used a single question comprised of 13 images of common cutaneous lesions and two pictures of BACL, for a total of 15 images. The images shown to respondents can be seen in the Supplemental material. BACL images were used from previous research with the author's permission, and images of other dermatological phenomena were taken from DermNet and used in accordance with their free use license (5).^1^, ^2^, ^3^, ^4^, ^5^, ^6^, ^7^, ^8^, ^9^, ^10^, ^11^, ^12^, ^13^ Participants were asked to select images that were representative of cutaneous lesions they had previously identified on their own bodies. If they selected BACL images, they were considered to be in the suspected *Bartonella* infection group. If they did not select a *Bartonella*-associated image, they were considered to be in the control group.

For the primary outcomes associated with neuropsychiatric symptomology, the General Anxiety Disorder-7 (GAD-7) was used for anxiety symptom severity. The GAD-7 has a range of 0–21 with cutoffs for 4 levels of symptom severity: 0–4: minimal, 5–9: mild, 10–14: moderate, and 15–21: severe (11). The Patient Health Questionnaire-9 (PHQ-9) was used for depression symptom severity. Scores on the PHQ-9 range from 0 to 27 with 5 categories of severity: 0–4: no depressive symptoms, 5–9: mild depressive symptoms, 10–14: moderate depressive symptoms, 15–19: moderately severe depressive symptoms, and 20–27: severe depressive symptoms (12). The Schizotypal Personality Questionnaire-Brief Revised Updated (SPQ-BRU) was used to measure symptoms of schizotypy. It includes three higher-order factors: interpersonal, cognitive perceptual, and disorganized. The SPQ-BRU ranges from 32 to 160, with higher scores indicating greater schizotypy (13).

## Results

### MTurk validation

**Figure 1:**
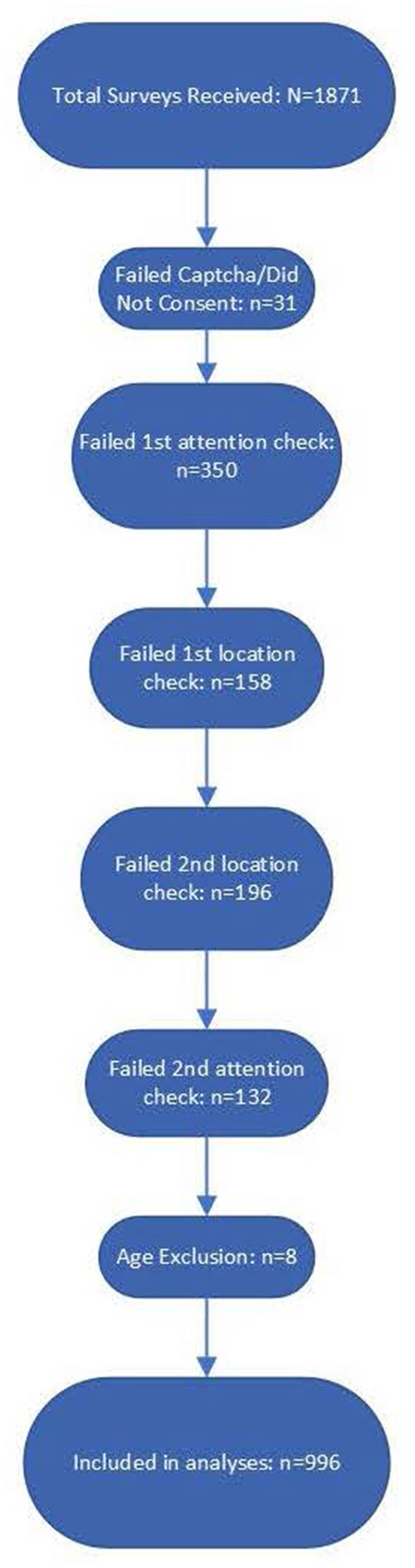
Flow diagram of survey responses from total received through data validation by each question. There were a total of five data validation questions which included captchas, location checks, and attention checks. There were also eight responses excluded because they self-reported an age under 18. Mturk requires Turkers to be atleast 18, so responses indicating an age under 18 were taken to be inattentive responses.

The total number of surveys received was *N* = 1,871. Following previous MTurk research protocols established for quality control, several attention checks and bot screening questions were embedded in the survey (10). Participants who failed to appropriately respond to attention-check questions had the survey terminated and were instructed to return the HIT. We implemented five attention check questions and also excluded surveys for which participants either did not consent or entered an age <18, which was an exclusionary criterion for participation. Although MTurk users are required to be 18 years of age to create an account, some responses indicated an age <18, which was interpreted either as inattention or that they had somehow bypassed the MTurk verification process and, as a result, were excluded. See Figure 1 for the flowchart of survey response rejection.

Out of 1,871 survey responses, 31 failed the Captcha question or did not consent. Notably, 350 participants did not successfully answer the first attention check, which instructed the respondent to select all possible choices despite what the question was asking. In addition, 158 respondents failed the first location check, which asked participants to identify the phone number they would call in an emergency; 196 respondents failed the second location check, which asked them to select the name of a vegetable in a photograph, which varies based on the part of the world one is in; 132 respondents failed the second attention check, which included a fictional creature in a list of selectable options; and eight respondents entered an age <18, which was required for participation. Following all attention, location, eligibility, and bot checks, there were *n* = 996 survey responses that were included in the analyses.

### Participant characteristics

Participants (*n* = 996) were primarily cisgender males (47.7%), cisgender females (41.5%), and white (81.5%) and had obtained at least an undergraduate degree (87.8%). The mean participant age was 37.5 years, with a minimum of 19 years and a maximum of 74 years (SD = 10.94). All participant characteristics can be found in Table 1.

**Table 1:** Participant characteristics.

		**No. (%)**
**Gender identity**		
	Cisgender male	475 (47.7)
	Transgender male	28 (2.8)
	Cisgender female	413 (41.5)
	Transgender female	34 (3.4)
	Gender non-conforming/gender questioning	8 (0.8)
	Prefer not to answer	30 (3.0)
	Other	7 (0.7)
**Race/ethnicity**		
	American Indian/Alaskan Native	6 (0.6)
	Asian	49 (4.9)
	Black/African American	65 (7.5)
	Latino/Latina	26 (2.6)
	Native Hawaiian/Pacific Islander	3 (0.3)
	White	811 (81.5)
	Multiracial/other	25 (2.5)
**Education**		
	Some high school	1 (0.1)
	High school/GED	43 (4.3)
	Vocational training	2 (0.2)
	Some college	51 (5.1)
	Associate's degree	25 (2.5)
	Bachelor's degree	677 (68.0)
	Some post-undergraduate	30 (3.0)
	Advanced degree	167 (16.8)

### Measures

**Figure 2:**
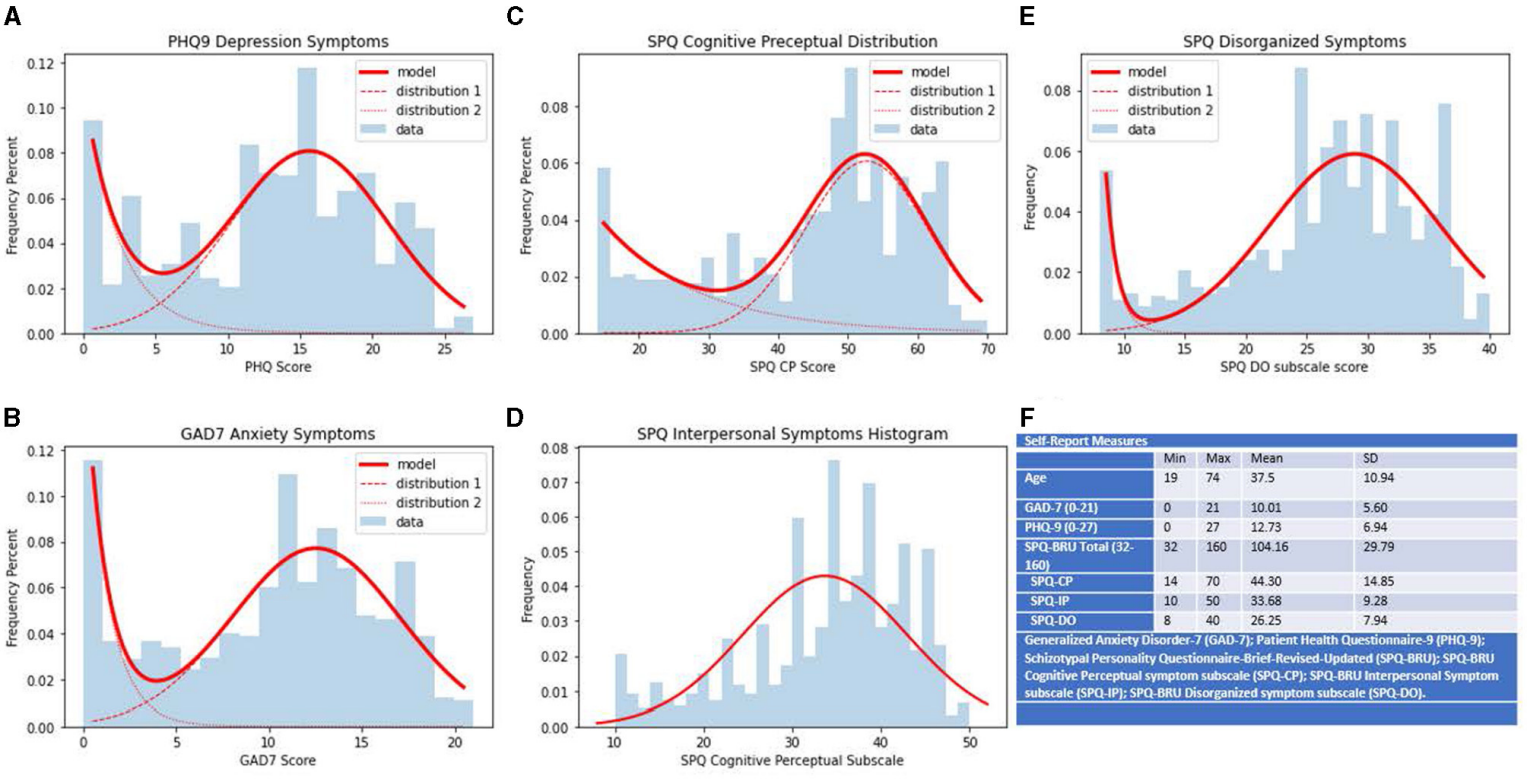
Ranges, distributions and summary statistics of self-report measures. Distributions of scores on collected self-report measures. Scores on measure are represented on the *x*-axis, frequency percents are represented on the *y*-axis. **(A)** PHQ-9 scores of self-report depression symptoms. **(B)** GAD-7 scores of self-report anxiety symptoms. **(C)** SPQ-BRU CP subfactor scores of self-report cognitive perceptual symptoms of schizotypy. **(D)** SPQ-BRU IP subfactor scores of self-report interpersonal symptoms of schizotypy. **(E)** SPQ-BRU DO subfactor scores of self-report disorganized symptoms of schizotypy. **(F)** Ranges and summary statistics of sample age and collected self-report measures.

The measures used in the study are expanded individually in the following paragraphs. The distributions and ranges for each measure can be found in Figure 2.

The Patient Health Questionnaire 9 (PHQ9) developed by Kroenke and Spitzer was administered to assess depressive symptoms (12). The sample had a minimum score of 0, a maximum score of 27, and a mean score of 12.73. The mean score for the sample meets the suggested cutoff criteria laid out by Kroenke et al. and lies within the range corresponding to moderate depressive symptoms (12). This finding is in line with previous MTurk research that found MTurk samples typically have higher scores on measures of depressive symptoms (14–17). The internal consistency for the PHQ9 within the sample was excellent, α = 0.923.

The GAD7 was used to assess anxiety symptoms. The 7-item measure had excellent internal consistency in our sample (α = 0.917), with a mean score of 10.01 and scores ranging from 0 to 21. Similarly to metrics on the PHQ9, the mean score for the sample was just above the clinical cutoff criteria outlined by the scale developers as indicative of a generalized anxiety disorder diagnosis and supported by other studies indicating MTurk samples exhibiting higher scores on anxiety measures (11, 14, 15).

The Schizotypal Personality Questionnaire Brief Revised Updated (SPQ-BRU) was administered to assess schizotypy. The Schizotypal Personality Questionnaire (SPQ) was developed by Raine but was consequently adapted to the SPQ-Brief (18, 19). The version used in the current study altered the SPQ-BR by making small wording changes to items to improve reliability (13). The SPQ-BRU is a 32-item measure scored on a 5-point Likert scale ranging from strongly disagree to agree for each item. Scores are further broken down into three higher-order factors and nine subfactors consistent with schizotypal spectrum theory. The mean score for the SPQ-BRU in the sample was 104.16 ± 29.79, with a minimum score of 32 and a maximum score of 160. The descriptive data for the higher-order subscales are given in Table 1.

### Relationship between BACL and psychiatric symptoms

**Figure 3:**
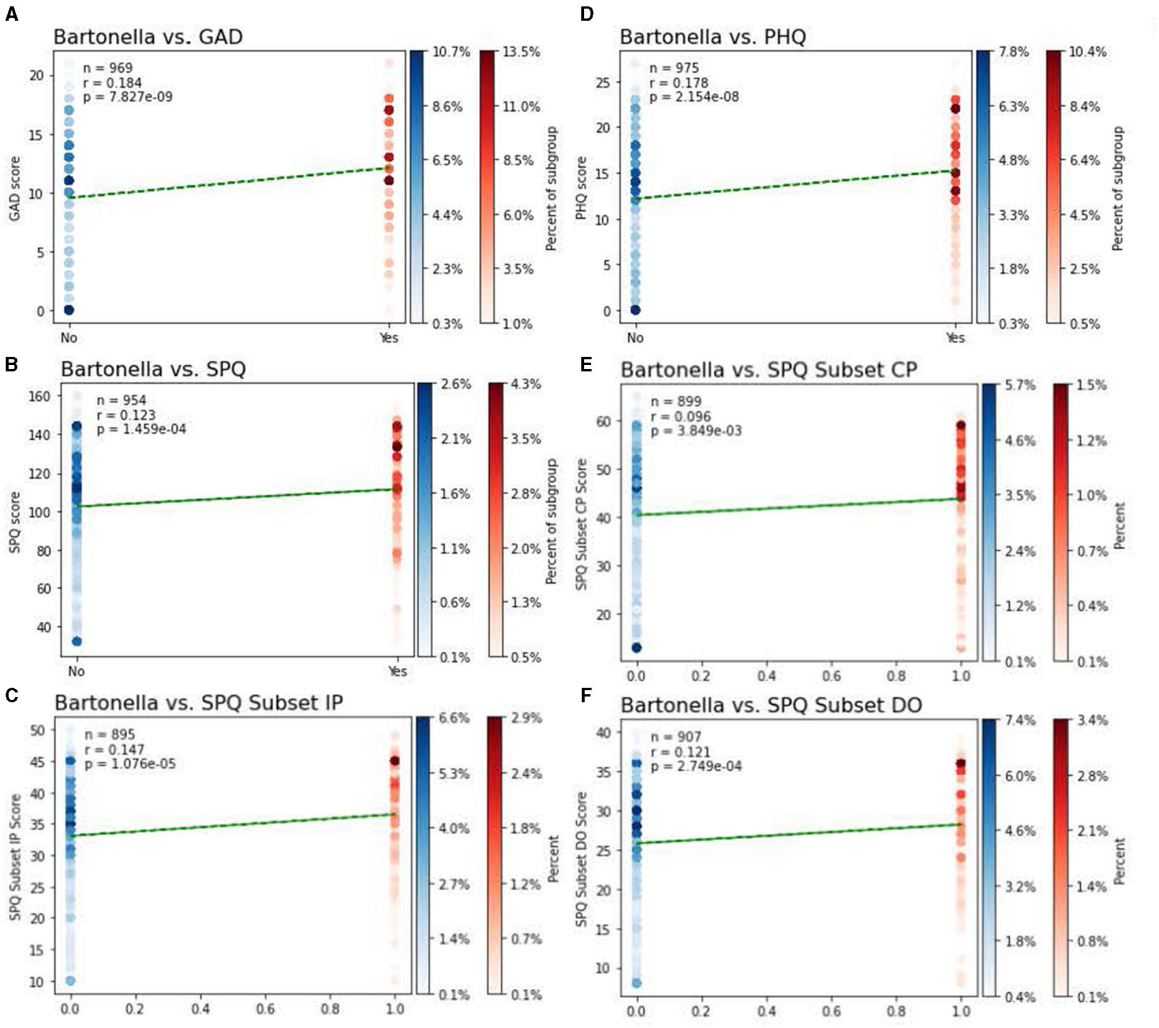
Relationships between the presence of BACL and psychiatric symptoms. Point biserial correlational analyses between the presence of BACL and psychiatric symptoms of **(A)** symptoms of anxiety as per the GAD-7, **(B)** symptoms of schizotypy as per the Schizotypal Personality Questionnaire Brief-Updated-Revised (SPQ-BRU) total score, **(C)** interpersonal symptoms of schizotypy as per the SPQ-BRU Interpersonal subfactor (items 7, 8, 9, 10, 11, 12, 17, 18, 19, 20), **(D)** symptoms of depression as per scores on the PHQ-9, **(E)** cognitive perceptual symptoms of schizotype as per the cognitive/perceptual subfactor (items 1, 2, 3, 4, 5, 6, 21, 22, 23, 24, 29, 30, 31, 32), **(F)** disorganized symptoms of schizotype as per the SPQ-BRU disorganized subfactor (items 13, 14, 15, 16, 25, 26, 27, 28). Individuals not selecting a BACL are represented in blue, individuals selecting a BACL images are represented in red. Shading of data points reflect the percentage of each group that reported symptom scores at that level, with darker points indicating a higher percentage of subject response at that level.

**Figure 4:**
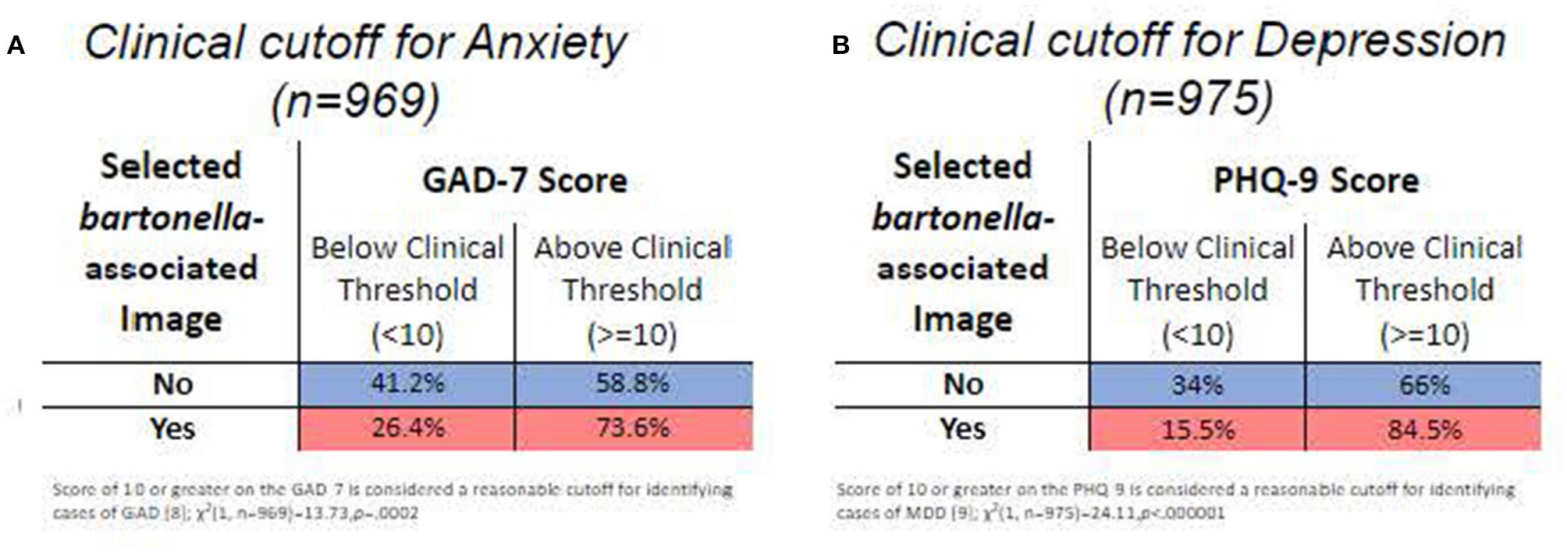
Chi square analyses with suggested clinical cutoffs for self-report anxiety and depression. Visualization of Chi square results. Percentages shaded in blue represent the percent of the sample that did not select as BACL image. Percentages shaded in red represent the percent of the sample that did select as BACL image. **(A)** Table comparing selection of BACL image with scores on GAD7 (Generalized Anxiety Disorder-7) split dichotomously by below or at/above suggested clinical cutoffs. **(B)** Table comparing selection of BACL image with scores on the PHQ-9 (Patient Health Questionnaire-9) split dichotomously by below or at/above suggested clinical cutoffs.

The primary outcome of the study was the relationship between the self-reported presence of BACL and neuropsychiatric symptoms, which was evaluated using point biserial correlation. A total of 19.5%, 95% CI: (0.1706, 0.2208) of participants reported BACL as something they had seen on their own body. The presence of BACL was positively associated with anxiety symptoms, *r*_(967)_ = 0.18, *p* < 0.0001, depression symptoms, *r*_(973)_ = 0.18, *p* < 0.0001, cognitive perceptual symptoms of schizotypy, *r*_(897)_ = 0.10, *p* < 0.01, interpersonal symptoms of schizotypy, *r*_(897)_ = 0.15, *p* < 0.0001, and disorganized symptoms of schizotypy, *r*_(905)_ = 0.12, *p* < 0.001. Visualizations of relationships are illustrated in Figures 3, 4. To further expand on this finding, we also performed chi-square analyses with clinical cutoffs for measures of anxiety, $\chi_{\left(1,\text{n}=969\right)}^{2}$ = 13.73, *p* < 0.001, and depression, $\chi_{\left(1,\text{n}=954\right)}^{2}$ = 24.11, *p* < 0.001. Scores on measures of both anxiety and depression reaching the clinical cutoff were significantly associated with selecting a BACL photograph. These findings suggest that individuals who selected BACL photographs also scored higher on self-reported measures of anxiety and depression. Furthermore, individuals selecting BACL photographs were more likely to score at or above the suggested clinical cutoffs on self-reported measures of anxiety and depression.

### Relationship between other cutaneous lesions and psychiatric symptoms

Point biserial correlational analyses were used to investigate the relationship between non-BACL and psychiatric symptoms. Several skin conditions were positively correlated with measures of anxiety, depression, and schizotypy. One non-BACL image relationship to psychiatric symptoms of note was actinic keratosis, which was positively and significantly correlated with anxiety, *r*_(969)_ = 0.191, *p* < 0.001, depression, *r*_(975)_ = 0.188, *p* < 0.001, and cognitive perceptual, *r*_(899)_ = 0.18, *p* < 0.001, interpersonal, *r*_(895)_ = 0.146, *p* < 0.001, and disorganized symptoms of schizotypy, *r*_(907)_ = 0.141, *p* < 0.001. However, associations between non-BACL and psychiatric symptoms were weak, and the large sample size resulted in the statistical significance of very weak associations. The correlational matrix and associated *p*-values are listed in Table 2. We also included two control photographs, one of an insect bite and an option to select “None”, indicating that none of the photographs represented a cutaneous lesion the participant had previously experienced. The relationship between psychiatric symptoms and insect bite was non-significant between measures of anxiety, *r*_(967)_ = −0.042, *p* > 0.05, depression, *r*_(973)_ = −0.028, *p* > 0.05, and interpersonal and disorganized symptoms of schizotypy, *r*_(893)_ = 0.005, *p* > 0.05 and *r*_(905)_ = 0.02, *p* > 0.05, respectively. Insect bite was negatively and significantly correlated with cognitive-perceptual symptoms of schizotypy, *r*_(896)_ = −0.067, *p* < 0.05. Relationships between not selecting any photographs and psychiatric symptoms were all negatively and significantly correlated: anxiety, *r*_(967)_ = −0.311, *p* < 0.001, depression, *r*_(973)_ = −0.333, *p* < 0.001, and cognitive-perceptual subscale, *r*_(897)_ = −0.309, *p* < 0.001, interpersonal subscale, *r*_(893)_ = −0.319, *p* < 0.001, and disorganized subscale, *r*_(905)_ = −0.324, *p* < 0.001. It is possible that this finding represents respondents who paid attention during the survey but wanted to finish the survey quickly, thus responding no to all questions. These results suggest that there may be a common, underlying mechanism germane to dermatological skin manifestations across diseases and disorders which drives the positive associations with psychiatric symptoms.

**Table 2:** Correlational matrix for skin lesions and psychiatric symptoms.

**Point-biserial correlation** ***r*****-values**						
	**GAD-7**	**PHQ-9**	**SPQ-Total**	**SPQ-CP**	**SPQ-IP**	**SPQ-DO**
**Skin condition image selected**						
BACL	0.184^**^	0.178^**^	0.123^**^	0.096^**^	0.147^**^	0.121^**^
Eczema	0.035	0.057	0.086	0.081^*^	0.09^*^	0.102^*^
Melasma	0.154^**^	0.153^**^	0.161^**^	0.174^**^	0.122^**^	0.153^**^
Acne	0.076^+^	0.066^+^	0.099^*^	0.066^+^	0.116^**^	0.118^**^
Cyst	0.151^**^	0.132^**^	0.109^**^	0.121^**^	0.081^+^	0.098^*^
Ichtyosis	0.128^**^	0.114^**^	0.122^**^	0.125^**^	0.109^*^	0.107^*^
Urticaria	0.162^**^	0.153^**^	0.109^**^	0.105^*^	0.094^*^	0.119^**^
Actinic keratosis	0.191^**^	0.188^**^	0.168^**^	0.18^**^	0.146^**^	0.141^**^
Measles	0.126^**^	0.117^**^	0.117^**^	0.103^*^	0.132^**^	0.106^*^
Vitiligo	0.14^**^	0.146^**^	0.128^**^	0.134^**^	0.103^*^	0.115^**^
Psoriasis	0.124^**^	0.12^**^	0.111^**^	0.099^*^	0.105^*^	0.125^**^
Insect bite	−0.042	−0.028	−0.026	−0.067^+^	−0.005	0.02
No skin manifestations	−0.311^**^	−0.333^**^	−0.327^**^	−0.309^**^	−0.319^**^	−0.324^**^

^+^*p* < 0.05; ^*^p < 0.01; ^**^p < 0.001.

GAD-7, generalized anxiety disorder-7; PHQ-9, Patient Health Questionnaire-9; SPQ, Schizotypal Personality Questionnaire; SPQ-CP, Schizotypal Personality Questionnaire-Cognitive Perceptual Subscale; SPQ-IP, Schizotypal Personality Questionnaire-Interpersonal subscale; SPQ-DO, Schizotypal Personality Questionnaire- Disorganized subscale; SPQ-BRU, Schizotypal Personality Questionnaire- Brief Revised Updated.

## Discussion

The purpose of this study was to investigate the potential relationship between cutaneous lesions associated with *Bartonella* infection and symptoms of anxiety, depression, and schizotypy in a large sample, broadening the scope of scientific inquiry into and awareness of the potential link between *Bartonella* infection and psychiatric symptoms by leveraging a potential component of *Bartonella* infection that is effectively represented in an online survey and easily disseminated to a large sample. Identifying BACL is only one component of a comprehensive diagnostic strategy. Verifying the diagnosis of *Bartonella* infection and establishing a causal link between Bartonella infection and psychiatric symptoms is beyond the scope of this investigation. However, awareness of BACL and its potential relationship with psychiatric symptoms can be a valuable component of a diagnostic strategy when biological verification methods are plausible.

We found that MTurk was a useful platform to quickly generate a large sample, receiving over 1,871 survey responses in approximately 5 h. Mturk data validation strategies from previous research (10) were employed to great effect, successfully eliminating inattentive responses, suspected bots, and additional location screening in a final analysis group of *n* = 996. The most effective validation question was an attention check question instructing individuals to select all the possible responses irrespective of what the question asked, which eliminated 350 responses. Secondary attention checks and location checks were also effective in screening out potentially low-quality data.

The main outcome of interest was the relationship between the presence of BACL and psychiatric symptoms of anxiety, depression, and schizotypy. Of the analysis sample (*n* = 996), 19.5% affirmed recognizing BACL as something they had seen on their own body. This prevalence is comparable to other research findings that reported *Bartonella* infection in populations across the United States (20–25). We found that self-reported symptoms of anxiety via scores on the GAD-7, depression via scores on the PHQ-9, and all three dimensions of schizotypy (cognitive/perceptual, interpersonal, and disorganized) were positively and significantly, albeit weakly, correlated with selecting images of BACL. These findings are in line with previous case studies that revealed a relationship between the onset of psychiatric symptoms and the diagnosis of *Bartonella spp*. infection, as well as the abatement of psychiatric symptoms following antibiotic treatment for *Bartonella* infection (3–5, 7, 26). In addition, chi-square analyses revealed that the presence of BACL was significantly related to self-reported scores of symptoms of anxiety and depression that fell above the suggested clinical cutoffs for both the GAD-7, with 73.6% of individuals affirming the presence of BACL on their body reporting a score of 10 or greater, and the PHQ-9, with 84.5% of individuals affirming the presence of BACL on their body reporting a score of 10 or greater. These findings are in line with previous Mturk research that has characterized Mturk samples as having higher than average levels of anxiety and depression (10, 17, 27). Findings are also congruent with previous case studies indicating an association between confirmed *Bartonella* infection, often with accompanying cutaneous lesions, and the onset or presence of severe symptoms of anxiety, psychosis, and depression (3–7).

We also found that symptoms of anxiety, depression, and schizotypy were also positively and significantly correlated with several other cutaneous lesions from other dermatological disorders. Images of actinic keratosis exhibited a positive relationship with symptoms of anxiety, depression, and schizotypy stronger than the relationship found between self-reported psychiatric symptoms and images of BACL. Actinic keratosis is a skin lesion that typically presents as a rough, scaly, dry patch of skin that may include discoloration (28). The leading cause of actinic keratosis is UV exposure, which disrupts the processes of cell growth and differentiation, leading to inflammation and an immune response (29). It is possible that the underlying mechanisms affecting the immune system in actinic keratosis may have similarities with the ways in which *Bartonella* species disrupt immune functioning, but these mechanisms are beyond the scope of this investigation and require further inquiry. While the images of these manifestations were used primarily to reduce false positives on the *Bartonella* images, the control images of insect bite and the option to select not having seen any of the images appear on one's own body were negatively correlated with symptoms of anxiety, depression, and schizotypy. Thus, we believe that perhaps the significant positive associations between a wide range of dermatological symptoms could be indicative of a common underlying biological process that is contributing to both dermatological and psychiatric symptoms.

There were a number of limitations present in this current investigation. First, while our data validation techniques were effective in eliminating a large number of inattentive and bot responses, further validation techniques can be instituted on a crowdsourcing platform such as MTurk. For example, the elimination of surveys completed too quickly would be a useful metric to ensure the quality of data and protect against submission times that took less time than it would to just read the survey questions in full. We did not implement this screening tool on the front end, so workers completing the surveys too fast were not warned ahead of time that their work may be rejected for responses that appeared too fast. Surveys that correctly answered all data fidelity questions were considered valid responses.

Second, participants were asked to self-identify different images of cutaneous lesions that they had seen on their own bodies as a proxy for identifying dermatological symptoms. The limitations of this are multi-faceted. It is possible that participants had difficulty differentiating between lesions seen on their own bodies, as represented by the images shown in our study. Individual participant memory and the extent to which they paid previous attention to skin lesions may have also affected respondents' ability to accurately select images of lesions seen on their own bodies. Diagnoses of cutaneous lesions in the participants by medical professionals would have likely exhibited substantially higher fidelity. In addition, identifying a lesion associated with a particular skin disorder is not indicative of a diagnosis. While the rash associated with Bartonellosis can be distinctive, appearing in long, thick striae or vertical red lesions with undulating borders and did not have similarity to the other images of skin disorders, we are not currently aware of any investigation that has been done on separating visual differences in causes of *Bartonella* infection striae from other medical conditions, and a verified diagnosis of *Bartonella* infection was beyond the scope of this study. Future studies should use a more definitive metric for tracking the presence or diagnosis of *Bartonella* infection. Furthermore, we did not assess the temporal characteristics of when the lesion appeared and psychiatric symptom onset, so we cannot be positive that the existence of skin lesions was in any way connected with psychiatric symptoms. Finally, all associations between the selection of BACL images and psychiatric symptoms were weak associations. Thus, we cannot rule out the possibility of a lack of a clinically meaningful association. However, our findings are still valuable in highlighting the potential link between *Bartonella* infection and psychiatric symptoms to raise awareness among clinicians. In many of the previously reported case studies and series, more standard treatments were employed to treat psychiatric symptomology with no benefit for the patient until *Bartonella* infection was diagnosed and antibiotics prescribed, resulting in the abatement of symptoms. Considering *Bartonella* infection when making diagnoses could expedite correct diagnoses and save patients from potential distress.

Third, the demographic characteristics of the sample and MTurk should be discussed. The sample was 81.5% white, 85% had at least a bachelor's degree, and all participants had access to the Internet. This is not representative of the general population of the United States, and the findings should not be applied as such (30, 31). Additionally, MTurk samples are well-documented as having higher than general population levels of anxiety and depression (15–17, 32). This should be taken into account when interpreting the findings of the association between the identification of BACL and psychiatric symptoms. Baseline levels of anxiety and depression symptoms in an MTurk population may contribute to or account for the relationship found between BACL and psychiatric symptoms. However, we hypothesize that the large percentage of participants who identified BACL as something on their body also have scores at or above the suggested clinical cutoffs, which could be important in elucidating the relationship between *Bartonella* infection and psychiatric symptoms.

## Conclusion

The current study broadens the link between the presence of BACL and the presence of psychiatric symptoms of anxiety, depression, and schizotypy and extends a potential relationship beyond the small sample sizes of previous case studies and case series. Scores at or above the suggested clinical cutoffs for anxiety and depression within a large percentage of the BACL self-identifying group suggest that, despite higher than general US population levels of anxiety and depression present in MTurk populations, an association may exist between *Bartonella* infection and psychiatric symptoms and requires further investigation. Data quality validation is a necessary component of using MTurk as a data collection platform, and the techniques used in this investigation were effective in improving the final analysis data quality. Future studies should expand the methods of data quality validation to include time limits on survey submission and should also pursue more accurate metrics for *Bartonella* and other dermatological infection diagnostic markers. More definitive diagnostic markers, such as PCR assays, should be used to confirm the actual presence of a given illness such as Bartonellosis and strengthen any potential relationship that may be found.

## Data availability statement

The raw data supporting the conclusions of this article will be made available by the authors, without undue reservation.

## Ethics statement

The studies involving humans were approved by University of North Carolina at Chapel Hill Biomedical Institutional Review Board. The studies were conducted in accordance with the local legislation and institutional requirements. The participants provided their written informed consent to participate in this study. Written informed consent was obtained from the individual(s) for the publication of any potentially identifiable images or data included in this article.

## Author contributions

ZS wrote the draft of the manuscript. FF provided mentorship and edits to the manuscript. SK was involved in the analysis and provided edits and figures and tables. All authors contributed to the article and approved the submitted version.

## Footnotes

^1^Acne. https://dermnetnz.org/assets/Uploads/acne/keratosis-pilaris/3016__WatermarkedWyJXYXRlcm1hcmtlZCJd.jpg.

^2^Acne. https://dermnetnz.org/assets/Uploads/acne/acne-back/142__WatermarkedWyJXYXRlcm1hcmtlZCJd.jpg.

^3^Melasma Image. https://dermnetnz.org/assets/Uploads/colour/chloas3__WatermarkedWyJXYXRlcm1hcmtlZCJd.jpg.

^4^Actinic Keratosis Image. https://dermnetnz.org/assets/Uploads/lesions/ak-hands/411__WatermarkedWyJXYXRlcm1hcmtlZCJd.jpg.

^5^Cyst Image. https://www.dermis.net/bilder/CD079/550px/img0093.jpg.

^6^Dermatitis Image. https://dermnetnz.org/assets/Uploads/dermatitis/atopic41__WatermarkedWyJXYXRlcm1hcmtlZCJd.jpg.

^7^Eczema Image. https://dermnetnz.org/assets/Uploads/dermatitis/flexural-eczema21__WatermarkedWyJXYXRlcm1hcmtlZCJd.jpg.

^8^Ichthyosis Image. https://dermnetnz.org/assets/Uploads/scaly/ichthyosis-10__WatermarkedWyJXYXRlcm1hcmtlZCJd.jpg.

^9^Measels Image. https://www.dermis.net/dermisroot/en/11877/image.htm.

^10^Insect bite image. https://www.dermis.net/bilder/CD033/550px/img0099.jpg.

^11^Psoriasis Image. https://dermnetnz.org/assets/Uploads/scaly/w/facial-psoriasis13__WatermarkedWyJXYXRlcm1hcmtlZCJd.jpg.

^12^Urticaria Image. https://dermnetnz.org/assets/Uploads/urticaria-301__WatermarkedWyJXYXRlcm1hcmtlZCJd.JPG.

^13^Vitiligo Image. https://dermnetnz.org/assets/Uploads/colour/vitiligo-21__WatermarkedWyJXYXRlcm1hcmtlZCJd.jpg.
