# Supplementary figures and images for: Crowd-sourced investigation of a potential relationship between Bartonella-associated cutaneous lesions and neuropsychiatric symptoms
Source: Front Psychiatry. 2023 Oct 24;14:1244121. doi: 10.3389/fpsyt.2023.1244121 (PMC10628448; doi:10.3389/fpsyt.2023.1244121)

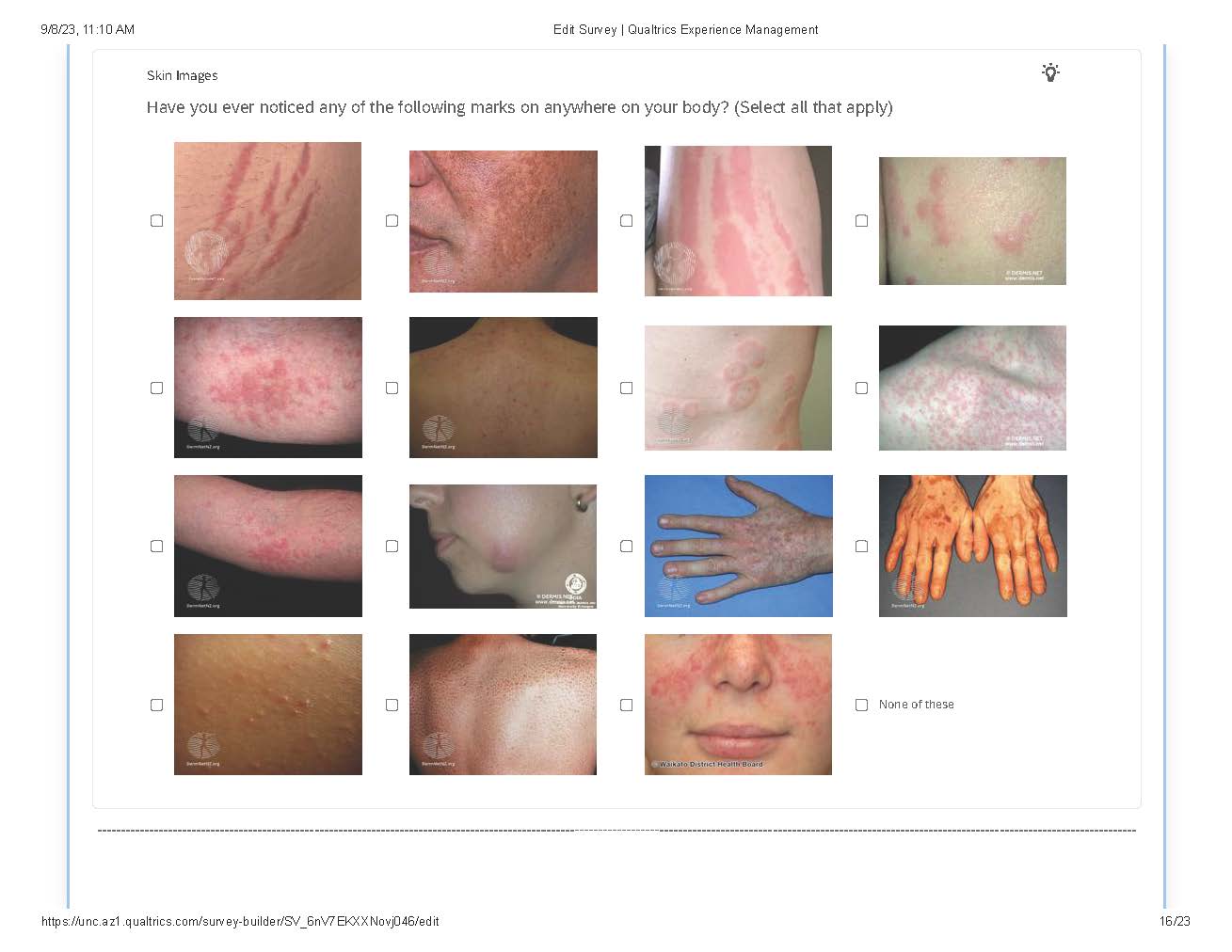

Supplement: Supplementary file 1 [file Image_1.JPEG]
